# Supplementary material for: A Multifaceted benchmarking of synthetic electronic health record generation models
Source: Nat Commun. 2022 Dec 9;13:7609. doi: 10.1038/s41467-022-35295-1 (PMC9734113; doi:10.1038/s41467-022-35295-1)
Supplement: Supplementary file 1 — Supplementary Information [file 41467_2022_35295_MOESM1_ESM.pdf]

Supplementary Information

A Multifaceted Benchmarking of Synthetic Electronic Health Record Generation Models

Authors:
Chao Yan[1], Yao Yan[2], Zhiyu Wan[1], Ziqi Zhang[3], Larsson Omberg[2], Justin Guinney[4,5], Sean D. Mooney[4,*], Bradley A. Malin[1,3,6,*]

Affiliations:
[1]Department of Biomedical Informatics, Vanderbilt University Medical Center, Nashville, TN, USA
[2]Sage Bionetworks, Seattle, WA, USA
[3]Department of Computer Science, Vanderbilt University, Nashville, TN, USA
[4]Department of Biomedical Informatics and Medical Education, University of Washington, Seattle, WA, USA
[5]Tempus Labs, Chicago, IL, USA
[6]Department of Biostatistics, Vanderbilt University, Nashville, TN, USA

These authors contributed equally: Chao Yan, Yao Yan, Zhiyu Wan.
These authors jointly supervised this work: Sean D. Mooney, Bradley A. Malin.

Corresponding authors:
Sean D. Mooney, Ph.D.
Email: sdmooney@uw.edu
Address: 850 Republican St, Seattle, WA, USA, 98109

Bradley A. Malin, Ph.D.
Email: b.malin@vumc.org
Address: Suite 1475, 2525 West End Ave, Nashville, TN, USA, 37203

Supplementary A: Comparison of synthesis paradigms

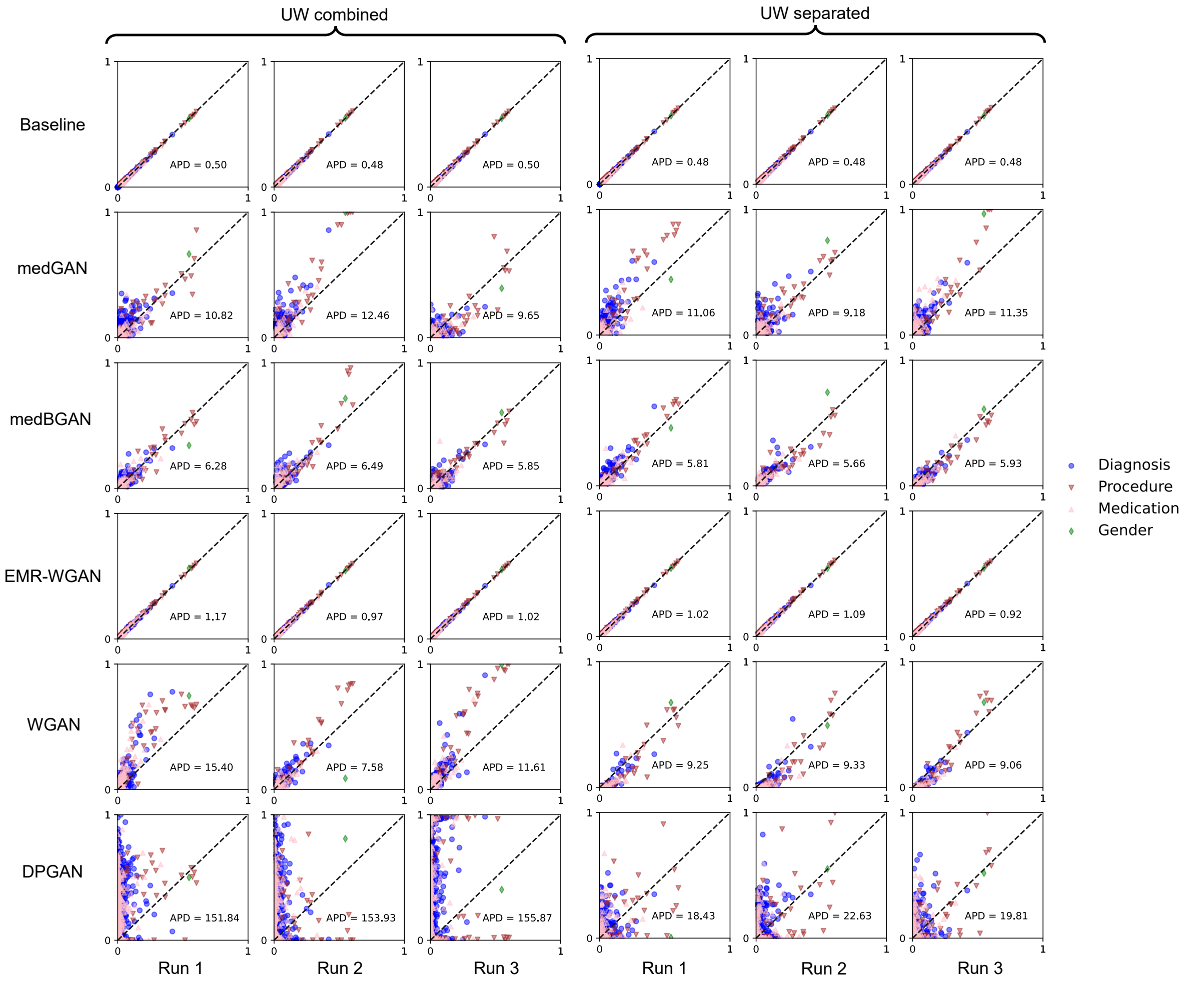

Supplementary Figure 1. **Dimension-wise distribution using a combined synthesis paradigm and a separated synthesis paradigm for the UW dataset**. Here, the x- and y-axes correspond to the prevalence of a feature in real and synthetic data, respectively. The results for three independently generated synthetic datasets are shown for each candidate model. Feature dots on the dashed diagonal line correspond to the perfect replication of prevalence. UW: The University of Washington.

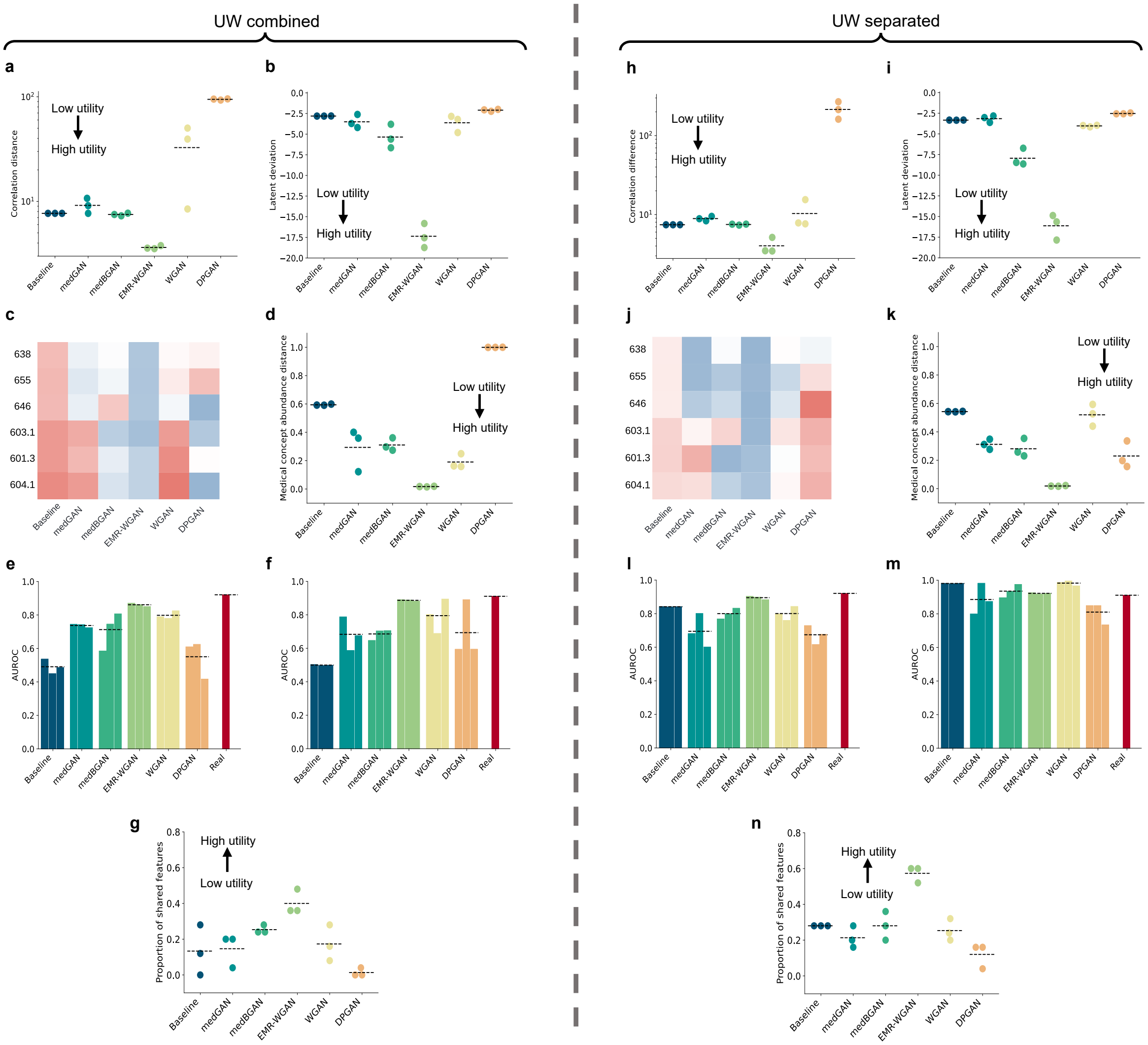

Supplementary Figure 2. **Data utility (except for dimension-wise distribution) from using separated synthesis paradigm (a-g) and combined synthesis paradigm (h-n) for the UW datasets. a**,**h** Column-wise correlation. **b**,**i** Latent cluster analysis. **c**,**j** Clinical knowledge violation for gender-specific phecodes. **d**,**k** Medical concept abundance. **e**,**l** TSTR Model performance for training on synthetic data. **f**,**m** TRTS Model performance. **g**,**n** The proportion of top *k* features in common (25 for UW). The heatmaps correspond to the ratio of clinical knowledge violations in gender (blue = low value; red = high value). A dashed line indicates the mean value across three synthetic datasets. (Phecode definitions: 625: Symptoms associated with female genital organs; 614: Inflammatory diseases of female pelvic organs; 792: Abnormal Papanicolaou smear of cervix and cervical HPV; 796: Elevated prostate-specific antigen; 601: Inflammatory diseases of prostate; 185: Prostate cancer; 638: Other high-risk pregnancy; 655: Known or suspected fetal abnormality; 646: Other complications of pregnancy NEC; 603.1: Hydrocele; 601.3: Orchitis and epididymitis; 604.1: Redundant prepuce and phimosis/BXO)

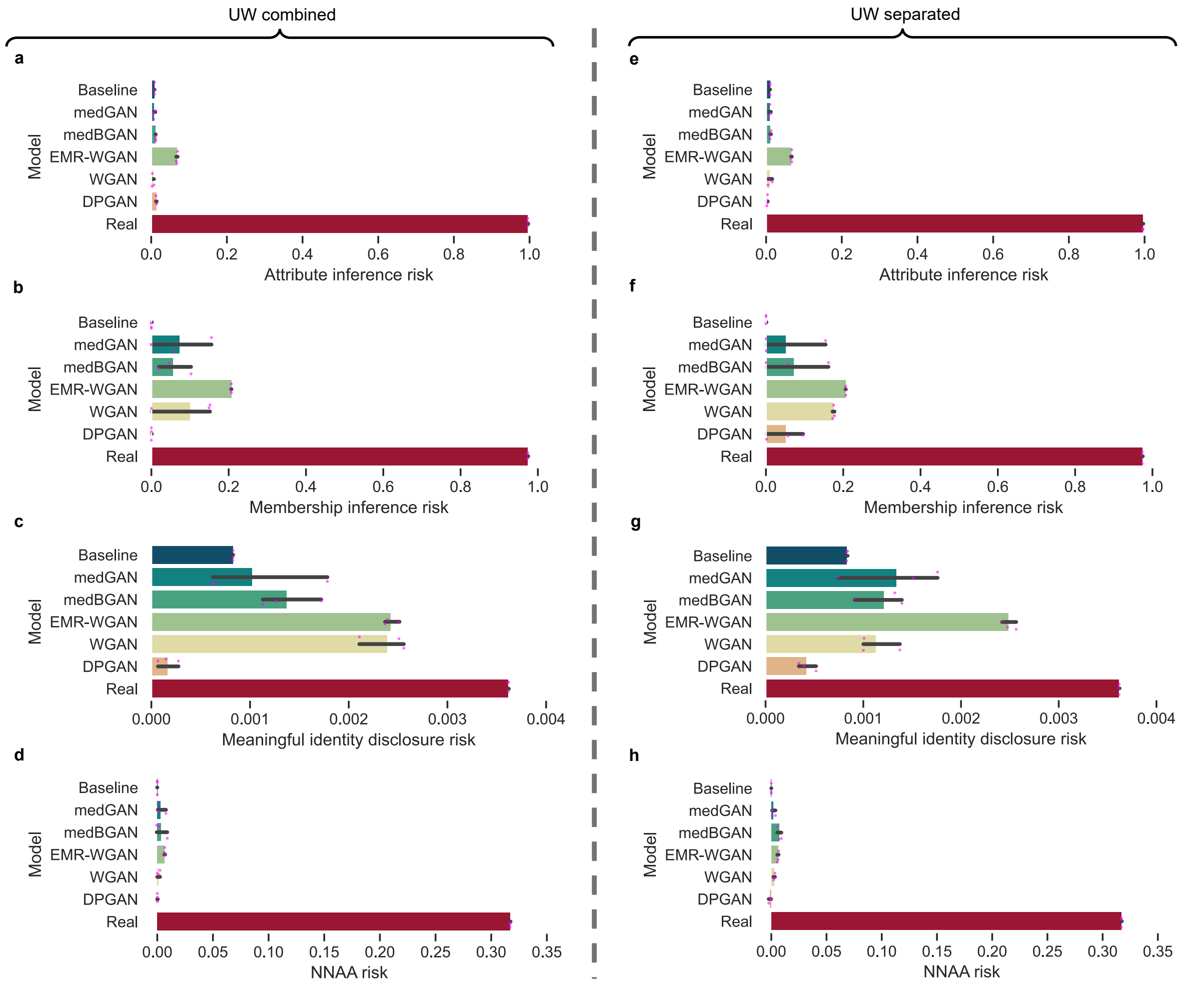

Supplementary Figure 3. **Average privacy risks (n=3) of the synthetic datasets generated for the UW synthetic data using combined synthesis paradigm (a-d) and separated synthesis paradigm (e-h)**. **a**,**e** Attribute inference risk. **b**,**f** Membership inference risk. **c**,**g** Meaning identity disclosure risk. **d**,**h** Nearest neighbor adversarial accuracy (NNAA) risk. The risks associated with the real data are shown in the bottom red bars. The 95% confidence intervals are marked as thin horizontal black lines.

Supplementary Table 1. **The rank-derived scores for models with respect to the individual metrics using UW synthetic data generated under the separated synthesis paradigm**. The best (i.e., lowest rank) and worst scores for each metric are marked as bold.

Metric	Model					
	**Baseline**	**medGAN**	**medBGAN**	**EMR-WGAN**	**WGAN**	**DPGAN**
Dimension-wise distribution	**2.0**	13.3	8.0	5.0	11.7	**17.0**
Column-wise correlation	6.0	13.0	7.0	**2.0**	12.0	**17.0**
Latent cluster analysis	12.0	13.0	5.0	**2.0**	8.0	**17.0**
Clinical knowledge violation	14.0	8.0	6.0	**2.0**	10.0	**17.0**
Medical concept abundance	**16.0**	9.3	8.3	**2.0**	15.0	6.3
Model performance (TRTS)	4.8	11.5	9.7	11.0	**3.7**	**16.3**
Model performance (TSTR)	6.0	14.0	10.2	**2.0**	9.2	**15.7**
Feature selection	8.0	12.3	8.3	**2.0**	9.7	**16.7**
Attribute inference risk	9.2	8.3	12.2	**17.0**	8.3	**2.0**
Member inference risk	**3.0**	5.7	8.7	**17.0**	14.0	8.7
Meaningful identity disclosure risk	6.0	11.0	10.7	**17.0**	10.3	**2.0**
NNAA risk	**5.0**	9.0	**16.2**	14.8	10.0	2.0

Supplementary Table 2. **Overall rank of generative models for the use cases in the Model recommendation phase**. Model ranks were based on the benchmarking framework scores (in parenthesis). The fact that medGAN and DPGAN have the same score in the System Development use case is due to precision loss instead of an actual tie.

**Use case**	**Dataset**	**Final ranks of models**					
		**1**	**2**	**3**	**4**	**5**	**6**
**Education**	UW separated	EMR-WGAN (5.6)	Baseline (7.6)	medBGAN (8.3)	medGAN (11.1)	WGAN (11.2)	DPGAN (13.2)
**Medical AI Development**	UW separated	EMR-WGAN (6.5)	Baseline (7.0)	medBGAN (9.8)	WGAN (10.1)	medGAN (11.6)	DPGAN (12.1)
**System Development**	UW separated	Baseline (7.4)	DPGAN (8.7)	EMR-WGAN (9.7)	medBGAN (9.9)	medGAN (10.1)	WGAN (11.3)

Supplementary Table 3. **Rank-derived scores for the UW synthetic datasets considering both the combined synthesis paradigm and separated synthesis paradigm**. "_com" and "_sep" are used as suffix to denote the combined synthesis paradigm and the separated synthesis paradigm, respectively. The best (i.e., lowest rank) and worst scores for each metric are in bold font.

	**Baseline_com**	**Baseline_sep**	**medGAN_com**	**medGAN_sep**	**medBGAN_com**	**medBGAN_sep**	**EMR-WGAN_com**	**EMR-WGAN_sep**	**WGAN_com**	**WGAN_sep**	**DPGAN_com**	**DPGAN_sep**
Dimension-wise distribution	4.0	**3.0**	26.0	24.7	16.7	14.3	9.8	9.2	25.7	21.7	**35.0**	32.0
Column-wise correlation	18.0	10.0	22.7	24.0	13.0	11.7	4.0	**3.0**	27.3	21.3	32.0	**35.0**
Latent cluster analysis	28.0	21.0	20.3	23.0	12.7	8.0	**2.7**	4.3	20.0	15.0	**35.0**	32.0
Clinical knowledge violation	31.7	28.0	20.7	17.3	11.0	13.7	**2.3**	5.3	24.7	21.0	11.7	**34.7**
Medical concept abundance	**31.3**	28.0	17.7	18.0	18.3	15.7	3.0	4.0	10.7	27.7	**35.0**	12.7
Model performance (TRTS)	**35.0**	4.8	28.7	15.2	27.7	9.7	16.7	11.0	21.3	**3.7**	26.3	22.0
Model performance (TSTR)	**34.0**	9.0	23.3	23.7	22.0	15.2	5.0	**2.0**	15.7	14.2	31.3	26.7
Feature selection	25.5	12.5	25.3	20.3	16.2	13.5	5.3	**2.0**	23.0	16.0	**34.0**	28.3
Attribute inference risk	13.0	17.2	12.0	16.3	23.0	21.8	**34.2**	32.8	5.0	15.7	27.7	**3.3**
Membership inference risk	**6.5**	**6.5**	17.5	12.7	18.3	19.7	**34.7**	32.3	20.0	29.0	**6.5**	18.3
Meaningful identity disclosure risk	12.0	13.0	14.0	19.7	21.3	20.0	30.8	**32.8**	32.3	19.0	**2.0**	5.0
NNAA risk	9.3	12.3	22.3	19.3	19.0	**31.5**	31.0	28.2	16.0	22.7	8.0	**2.3**

Supplementary Table 4. **Overall rank of generative models for the use cases in the Model recommendation phase using UW data under both separated and combined synthesis paradigms**. Model ranks were based on the benchmarking framework scores (in parenthesis). "_com" and "_sep" are used as suffix to denote the combined synthesis paradigm and the separated synthesis paradigm, respectively.

Use case	Final ranks of models					
	1	2	3	4	5	6
**Education**	EMR-WGAN_sep (10.7)	EMR-WGAN_com (10.9)	Baseline_sep (13.8)	medBGAN_sep (15.4)	medBGAN_com (16.2)	Baseline_com (18.0)
**Medical AI Development**	EMR-WGAN_sep (11.5)	Baseline_sep (12.3)	EMR-WGAN_com (13.2)	medBGAN_sep (16.9)	WGAN_sep (18.1)	WGAN_com (18.8)
**System Development**	Baseline_sep (14.0)	Baseline_com (15.9)	DPGAN_sep (16.6)	medBGAN_com (18.4)	EMR-WGAN_sep (18.4)	medBGAN_sep (18.6)

Use case	Final ranks of models					
	7	8	9	10	11	12
**Education**	medGAN_sep (20.9)	WGAN_sep (21.1)	medGAN_com (21.5)	WGAN_com (21.9)	DPGAN_sep (25.5)	DPGAN_com (26.0)
**Medical AI Development**	medBGAN_com (19.1)	medGAN_sep (20.7)	medGAN_com (21.2)	DPGAN_sep (21.6)	Baseline_com (23.3)	DPGAN_com (25.3)
**System Development**	EMR-WGAN_com (19.0)	WGAN_com (19.1)	medGAN_sep (19.2)	medGAN_com (19.3)	WGAN_sep (21.7)	DPGAN_com (21.8)

Supplementary B: Detailed results for evaluation metrics.

Supplementary Table 5. **Results for dimension-wide distribution**. The value is absolute prevalence rate difference (APD) for UW and combination of APD and average of the variable-wise Wasserstein distances (AWD) for VUMC.

	Run	Baseline	medGAN	medBGAN	EMR-WGAN	WGAN	DPGAN
**UW (combined)**	1	0.496	10.818	6.283	1.165	15.397	151.839
	2	0.477	12.464	6.488	0.969	7.581	153.927
	3	0.497	9.654	5.845	1.018	11.606	155.866
**UW (separated)**	1	0.481	11.056	5.812	1.018	9.250	18.426
	2	0.481	9.178	5.658	1.091	9.330	22.635
	3	0.480	11.350	5.934	0.920	9.065	19.810
**VUMC**	1	2.487	16.638	11.845	3.122	4.703	16.231
	2	2.481	18.024	11.795	4.557	3.625	13.979
	3	2.479	14.047	12.263	5.686	3.161	14.152

Supplementary Table 6. **Results for column-wise correlation**.

	Run	Baseline	medGAN	medBGAN	EMR-WGAN	WGAN	DPGAN
**UW (combined)**	1	7.686	9.106	7.497	3.573	50.189	95.029
	2	7.685	10.703	7.713	3.534	8.462	93.027
	3	7.686	7.684	7.281	3.780	39.406	95.493
**UW (separated)**	1	7.429	8.863	7.568	5.141	7.843	268.623
	2	7.433	8.340	7.553	3.481	15.454	161.342
	3	7.437	9.495	7.352	3.456	7.632	213.637
**VUMC**	1	20.277	19.522	19.184	11.884	14.569	18.835
	2	20.275	19.138	18.117	12.008	12.432	18.186
	3	20.276	18.500	18.195	12.441	11.437	18.977

Supplementary Table 7. **Latent deviation for latent cluster analysis**.

	**Run**	**Baseline**	**medGAN**	**medBGAN**	**EMR-WGAN**	**WGAN**	**DPGAN**
**UW (combined)**	1	-2.816	-3.712	-5.602	-18.736	-3.216	-2.000
	2	-2.815	-2.614	-3.804	-17.547	-4.810	-2.235
	3	-2.800	-4.196	-6.650	-15.822	-2.848	-2.059
**UW (separated)**	1	-3.345	-3.025	-8.638	-15.647	-3.989	-2.568
	2	-3.332	-3.613	-6.735	-14.880	-4.151	-2.465
	3	-3.327	-2.828	-8.459	-17.848	-3.962	-2.568
**VUMC**	1	-2.485	-2.592	-3.874	-16.860	-10.540	-2.680
	2	-2.486	-2.531	-12.437	-12.141	-10.067	-2.588
	3	-2.484	-6.846	-8.888	-9.344	-11.594	-6.132

Supplementary Table 8. **Clinical knowledge violation results for UW synthetic data using the combined synthesis paradigm**.

UW combined	Run	Violation on female-only diseases			Violation on male-only diseases		
		**638**	**655**	**646**	**603.1**	**601.3**	**604.1**
**Baseline**	1	43.97%	43.95%	45.00%	52.95%	54.73%	57.76%
	2	44.27%	45.15%	44.32%	52.53%	53.79%	57.51%
	3	43.17%	46.31%	44.41%	57.11%	54.14%	59.17%
**medGAN**	1	8.74%	2.45%	9.49%	46.88%	21.72%	53.85%
	2	0.04%	0.05%	0.00%	99.60%	100.00%	100.00%
	3	50.22%	47.37%	47.33%	0.00%	13.15%	7.14%
**medBGAN**	1	33.72%	35.95%	47.56%	0.46%	4.76%	5.45%
	2	10.80%	9.64%	12.59%	15.71%	33.33%	25.81%
	3	25.30%	17.62%	60.88%	8.70%	17.24%	14.29%
**EMR-WGAN**	1	4.54%	4.95%	5.90%	2.85%	9.25%	8.75%
	2	5.33%	4.59%	5.15%	6.27%	6.72%	14.44%
	3	7.21%	6.56%	5.70%	0.74%	12.57%	5.40%
**WGAN**	1	2.26%	2.04%	7.26%	68.18%	88.75%	92.03%
	2	75.00%	87.84%	57.14%	4.60%	4.32%	6.25%
	3	0.00%	0.00%	0.00%	87.34%	80.22%	89.29%
**DPGAN**	1	15.09%	50.00%	0.00%	18.82%	33.56%	NAN
	2	17.30%	19.08%	NAN	0.00%	0.00%	0.00%
	3	51.39%	60.10%	NAN	0.00%	40.31%	0.00%

Supplementary Table 9. **Clinical knowledge violation results for UW synthetic data using the separated synthesis paradigm**.

UW separated	Run	Violation on female-only diseases			Violation on male-only diseases		
		**638**	**655**	**646**	**603.1**	**601.3**	**604.1**
**Baseline**	1	45.44%	45.17%	45.30%	56.00%	55.30%	50.00%
	2	45.17%	44.20%	43.58%	51.47%	56.04%	52.71%
	3	44.19%	45.95%	45.35%	54.72%	51.55%	51.25%
**medGAN**	1	18.76%	19.26%	44.44%	25.35%	13.35%	0.00%
	2	0.65%	1.60%	0.17%	0.00%	92.11%	100.00%
	3	1.25%	0.59%	0.27%	99.59%	95.83%	NaN
**medBGAN**	1	60.35%	29.56%	38.61%	19.31%	2.20%	4.96%
	2	15.59%	4.55%	2.23%	70.00%	4.36%	29.72%
	3	31.90%	6.57%	23.65%	71.42%	11.54%	18.75%
**EMR-WGAN**	1	6.49%	6.06%	6.48%	8.42%	8.55%	9.46%
	2	3.94%	4.62%	4.84%	3.89%	8.10%	13.89%
	3	5.10%	8.61%	4.88%	6.09%	12.50%	10.34%
**WGAN**	1	30.00%	0.00%	21.21%	60.00%	44.44%	50.00%
	2	42.52%	36.67%	24.86%	34.62%	28.57%	30.70%
	3	41.55%	24.76%	33.33%	NaN	NaN	NaN
**DPGAN**	1	99.91%	99.62%	99.95%	0.10%	0.09%	0.11%
	2	0.00%	0.00%	92.41%	100.00%	100.00%	100.00%
	3	0.62%	NaN	66.67%	100.00%	87.47%	100.00%

Supplementary Table 10. **Clinical knowledge violation results for VUMC synthetic data**.

VUMC	Run	Violation on female-only diseases			Violation on male-only diseases		
		625	614	792	796	601	185
**Baseline**	1	49.05%	44.93%	49.11%	60.00%	52.04%	50.47%
	2	48.50%	42.33%	43.58%	60.29%	52.07%	60.00%
	3	44.72%	41.34%	45.71%	60.16%	56.64%	57.61%
**medGAN**	1	6.69%	0.00%	5.83%	NaN	NaN	100.00%
	2	50.00%	87.50%	36.48%	1.43%	0.00%	0.00%
	3	2.52%	0.00%	50.00%	0.00%	0.00%	NaN
**medBGAN**	1	5.55%	21.27%	36.50%	35.71%	0.00%	33.33%
	2	14.29%	31.86%	50.00%	0.00%	7.41%	0.00%
	3	4.88%	37.69%	25.00%	42.86%	40.00%	50.00%
**EMR-WGAN**	1	8.73%	9.35%	10.82%	20.77%	9.38%	16.50%
	2	2.89%	9.62%	11.59%	15.79%	17.84%	23.21%
	3	8.08%	11.72%	6.44%	21.94%	16.67%	13.79%
**WGAN**	1	1.35%	6.22%	8.60%	14.94%	0.00%	20.59%
	2	6.26%	4.96%	3.59%	3.48%	7.55%	10.20%
	3	8.77%	9.25%	8.56%	5.21%	2.82%	9.86%
**DPGAN**	1	20.00%	33.33%	20.83%	66.66%	0.00%	100.00%
	2	17.39%	NaN	33.33%	36.76%	NaN	52.94%
	3	13.04%	22.72%	23.39%	0.00%	NaN	66.66%

Supplementary Table 11. **Results for medical concept abundance metric.**

	**Run**	**Baseline**	**medGAN**	**medBGAN**	**EMR-WGAN**	**WGAN**	**DPGAN**
**UW (combined)**	1	0.592	0.122	0.297	0.019	0.163	1.000
	2	0.591	0.359	0.361	0.015	0.159	0.999
	3	0.599	0.400	0.274	0.017	0.249	0.999
**UW (separated)**	1	0.544	0.348	0.354	0.018	0.527	0.336
	2	0.542	0.277	0.258	0.017	0.593	0.198
	3	0.541	0.311	0.231	0.022	0.440	0.156
**VUMC**	1	0.742	0.124	0.205	0.038	0.105	0.194
	2	0.760	0.195	0.155	0.017	0.048	0.076
	3	0.757	0.144	0.091	0.034	0.054	0.137

Supplementary Table 12. **Model performance results for training on UW synthetic data generated using combined synthesis paradigm and testing on UW real data (TSTR)**. In contrast, training on 70% UW real data and testing on 30% UW real data lead to AUC 0.921 [0.916,0.926].

UW combined	Run_1	Run_2	Run_3	Overall
**Baseline**	0.537 [0.523,0.553]	0.449 [0.435,0.463]	0.486 [0.473,0.500]	0.491 [0.438,0.548]
**medGAN**	0.745 [0.733,0.757]	0.742 [0.731,0.753]	0.724 [0.713,0.735]	0.737 [0.716,0.754]
**medBGAN**	0.585 [0.571,0.599]	0.746 [0.734,0.757]	0.807 [0.797,0.817]	0.713 [0.575,0.814]
**EMR-WGAN**	0.870 [0.863,0.878]	0.862 [0.854,0.871]	0.851 [0.842,0.859]	0.861 [0.845,0.876]
**WGAN**	0.790 [0.778,0.801]	0.779 [0.768,0.790]	0.825 [0.816,0.834]	0.798 [0.771,0.832]
**DPGAN**	0.610 [0.596,0.625]	0.625 [0.614,0.636]	0.417 [0.405,0.429]	0.550 [0.408,0.634]

Supplementary Table 13. **Model performance results for training on UW synthetic data generated using separated synthesis paradigm and testing on UW real data (TSTR)**. In contrast, training on 70% UW real data and testing on 30% UW real data leads to AUC 0.921 [0.916,0.926].

UW separated	Run_1	Run_2	Run_3	Overall
**Baseline**	0.842 [0.833,0.851]	0.841 [0.831,0.850]	0.842 [0.833,0.851]	0.842 [0.832,0.851]
**medGAN**	0.682 [0.669,0.693]	0.801 [0.792,0.811]	0.602 [0.590,0.613]	0.695 [0.594,0.808]
**medBGAN**	0.769 [0.759,0.779]	0.799 [0.789,0.810]	0.833 [0.823,0.843]	0.800 [0.761,0.840]
**EMR-WGAN**	0.904 [0.898,0.910]	0.899 [0.892,0.905]	0.883 [0.876,0.890]	0.895 [0.878,0.908]
**WGAN**	0.799 [0.789,0.808]	0.760 [0.750,0.771]	0.843 [0.835,0.852]	0.801 [0.752,0.849]
**DPGAN**	0.729 [0.719,0.740]	0.617 [0.605,0.629]	0.677 [0.667,0.687]	0.674 [0.608,0.737]

Supplementary Table 14. **Model performance results for training on UW real data and testing on UW synthetic data generated using combined synthesis paradigm**. In contrast, training on 30% UW real data and testing on 70% UW real data lead to AUC 0.911 [0.907,0.915].

UW combined	Run_1	Run_2	Run_3	Overall
**Baseline**	0.503 [0.495,0.511]	0.497 [0.489,0.506]	0.498 [0.490,0.506]	0.500 [0.491,0.509]
**medGAN**	0.788 [0.781,0.795]	0.587 [0.579,0.594]	0.676 [0.668,0.684]	0.684 [0.581,0.793]
**medBGAN**	0.647 [0.640,0.655]	0.704 [0.697,0.711]	0.706 [0.698,0.713]	0.686 [0.642,0.712]
**EMR-WGAN**	0.891 [0.887,0.895]	0.889 [0.884,0.893]	0.881 [0.877,0.885]	0.887 [0.877,0.894]
**WGAN**	0.802 [0.794,0.810]	0.689 [0.681,0.697]	0.894 [0.890,0.899]	0.795 [0.683,0.898]
**DPGAN**	0.595 [0.591,0.599]	0.890 [0.888,0.893]	0.595 [0.587,0.605]	0.693 [0.589,0.892]

Supplementary Table 15. **Model performance results for training on UW real data and testing on UW synthetic data generated using separated synthesis paradigm**. In contrast, training on 30% UW real data and testing on 70% UW real data lead to AUC 0.911 [0.907,0.915].

UW separated	Run_1	Run_2	Run_3	Overall
**Baseline**	0.982 [0.980,0.983]	0.980 [0.978,0.981]	0.981 [0.980,0.983]	0.981 [0.979,0.983]
**medGAN**	0.800 [0.794,0.805]	0.982 [0.981,0.984]	0.874 [0.870,0.878]	0.885 [0.795,0.984]
**medBGAN**	0.896 [0.892,0.900]	0.934 [0.931,0.936]	0.975 [0.973,0.977]	0.935 [0.893,0.977]
**EMR-WGAN**	0.926 [0.923,0.929]	0.918 [0.915,0.921]	0.922 [0.919,0.925]	0.922 [0.916,0.928]
**WGAN**	0.987 [0.985,0.989]	0.996 [0.995,0.996]	0.966 [0.963,0.968]	0.983 [0.964,0.996]
**DPGAN**	0.849 [0.843,0.854]	0.848 [0.842,0.855]	0.734 [0.729,0.739]	0.810 [0.730,0.854]

Supplementary Table 16. **Model performance results for training on VUMC synthetic data and testing on VUMC real data**. In contrast, training on 70% VUMC real data and testing on 30% VUMC real data leads to AUC 0.802 [0.772,0.830].

VUMC	Run_1	Run_2	Run_3	Overall
**Baseline**	0.617 [0.582,0.652]	0.575 [0.539,0.613]	0.500 [0.462,0.539]	0.564 [0.470,0.643]
**medGAN**	0.675 [0.640,0.706]	0.696 [0.662,0.731]	0.559 [0.523,0.598]	0.643 [0.532,0.722]
**medBGAN**	0.567 [0.530,0.606]	0.558 [0.518,0.596]	0.656 [0.618,0.691]	0.594 [0.527,0.685]
**EMR-WGAN**	0.717 [0.685,0.748]	0.728 [0.695,0.760]	0.691 [0.657,0.725]	0.712 [0.665,0.753]
**WGAN**	0.635 [0.596,0.669]	0.646 [0.612,0.680]	0.733 [0.696,0.767]	0.671 [0.605,0.757]
**DPGAN**	0.629 [0.593,0.667]	0.685 [0.652,0.716]	0.661 [0.625,0.693]	0.658 [0.602,0.709]

Supplementary Table 17. **Model performance results for training on VUMC real data and test on VUMC synthetic data**. In contrast, training on 30% VUMC real data and testing on 70% VUMC real data lead to AUC 0.773 [0.752,0.796].

VUMC	Run_1	Run_2	Run_3	Overall
**Baseline**	0.508 [0.480,0.534]	0.502 [0.476,0.527]	0.499 [0.475,0.525]	0.503 [0.477,0.530]
**medGAN**	0.712 [0.689,0.734]	0.675 [0.653,0.696]	0.543 [0.518,0.571]	0.643 [0.524,0.728]
**medBGAN**	0.520 [0.495,0.546]	0.552 [0.532,0.573]	0.610 [0.583,0.637]	0.561 [0.502,0.630]
**EMR-WGAN**	0.691 [0.668,0.714]	0.724 [0.703,0.748]	0.634 [0.610,0.657]	0.683 [0.616,0.742]
**WGAN**	0.604 [0.581,0.629]	0.596 [0.571,0.620]	0.672 [0.649,0.695]	0.624 [0.577,0.689]
**DPGAN**	0.515 [0.492,0.537]	0.569 [0.544,0.594]	0.518 [0.493,0.542]	0.534 [0.494,0.586]

Supplementary Table 18. **Results for feature selection**.

	Run	Baseline	medGAN	medBGAN	EMR-WGAN	WGAN	DPGAN
**UW (combined)**	1	0.12	0.20	0.24	0.48	0.08	0.00
	2	0.28	0.04	0.28	0.36	0.16	0.00
	3	0.00	0.20	0.24	0.36	0.28	0.04
**UW (separated)**	1	0.28	0.16	0.36	0.52	0.32	0.16
	2	0.28	0.28	0.20	0.60	0.20	0.04
	3	0.28	0.20	0.28	0.60	0.24	0.16
**VUMC**	1	0.40	0.55	0.50	0.45	0.75	0.60
	2	0.45	0.45	0.50	0.50	0.55	0.55
	3	0.45	0.60	0.50	0.60	0.60	0.60

Supplementary Table 19. **Results for attribute inference risk**. *k* is the number of neighbors.

k	**Known features**	**Data**	**Run**	**Baseline**	**medGAN**	**medBGAN**	**EMR-WGAN**	**WGAN**	**DPGAN**	**Real**
**1**	**256**	**UW (combined)**	1	0.00875	0.01096	0.01167	0.06611	0.00213	0.01344	0.99531
			2	0.00865	0.00586	0.01166	0.06789	0.00319	0.01282	0.99531
			3	0.00881	0.00659	0.01191	0.06986	0.00733	0.01447	0.99531
		**UW (separated)**	1	0.01036	0.00689	0.01059	0.06611	0.01607	0.00193	0.99531
			2	0.01044	0.01263	0.01300	0.06779	0.00752	0.00393	0.99531
			3	0.01059	0.01008	0.01089	0.06782	0.00499	0.00432	0.99531
		**VUMC**	1	0.09637	0.08594	0.08748	0.14969	0.12798	0.08031	0.99593
			2	0.09687	0.08098	0.08511	0.14895	0.15233	0.10323	0.99593
			3	0.09693	0.08848	0.09358	0.15233	0.14810	0.10233	0.99593
**1**	**1024**	**UW (combined)**	1	0.00190	0.00064	0.00104	0.03075	0.00006	0.00163	0.99830
			2	0.00201	0.00059	0.00102	0.03272	0.00004	0.00229	0.99830
			3	0.00225	0.00071	0.00090	0.03253	0.00017	0.00224	0.99830
		**UW (separated)**	1	0.00211	0.00033	0.00081	0.03113	0.00213	0.00005	0.99830
			2	0.00204	0.00065	0.00082	0.03083	0.00030	0.00008	0.99830
			3	0.00231	0.00068	0.00108	0.03239	0.00017	0.00009	0.99830
		**VUMC**	1	0.19350	0.14313	0.15695	0.16574	0.13413	0.14044	0.99199
			2	0.19327	0.14264	0.14056	0.16882	0.17658	0.18659	0.99199
			3	0.19365	0.13977	0.15107	0.16250	0.17515	0.17253	0.99199
**10**	**256**	**UW (combined)**	1	0.00000	0.00274	0.00216	0.02295	0.00004	0.01373	0.03992
			2	0.00000	0.00121	0.00091	0.02368	0.00006	0.01275	0.03992
			3	0.00000	0.00032	0.00113	0.02606	0.00093	0.01445	0.03992
		**UW (separated)**	1	0.00000	0.00084	0.00045	0.02355	0.00160	0.00162	0.03992
			2	0.00000	0.00423	0.00144	0.02333	0.00130	0.00351	0.03992
			3	0.00000	0.00155	0.00135	0.02299	0.00115	0.00274	0.03992
		**VUMC**	1	0.08283	0.07314	0.05368	0.06544	0.07434	0.04725	0.13151
			2	0.08373	0.04698	0.05636	0.07087	0.07069	0.07335	0.13151
			3	0.08470	0.06802	0.05327	0.08959	0.06655	0.07682	0.13151

Supplementary Table 20. **Results for membership inference risk**. θ is the threshold for the Euclidean distance between two records.

θ	Data	Run	Baseline	medGAN	medBGAN	EMR-WGAN	WGAN	DPGAN	Real
**2**	**UW (combined)**	1	0.00000	0.15561	0.04662	0.20684	0.15168	0.00000	0.97374
		2	0.00000	0.00000	0.01961	0.20726	0.14929	0.00000	0.97374
		3	0.00000	0.06127	0.10261	0.20628	0.00012	0.00000	0.97374
	**UW (separated)**	1	0.00000	0.00000	0.00021	0.20601	0.17738	0.09607	0.97374
		2	0.00000	0.15449	0.05485	0.20619	0.17262	0.00159	0.97374
		3	0.00000	0.00000	0.16172	0.20678	0.17525	0.05718	0.97374
	**VUMC**	1	0.00000	0.22345	0.19321	0.27367	0.21279	0.00969	0.96428
		2	0.00000	0.08140	0.24246	0.27683	0.19567	0.20762	0.96428
		3	0.00000	0.19027	0.18375	0.27492	0.23326	0.00000	0.96428
**5**	**UW (combined)**	1	0.00000	0.25168	0.18518	0.29654	0.25083	0.00000	0.95967
		2	0.00000	0.00047	0.15972	0.29749	0.24937	0.00000	0.95967
		3	0.00000	0.19955	0.20351	0.29572	0.02717	0.00000	0.95967
	**UW (separated)**	1	0.00000	0.05134	0.13633	0.29583	0.27527	0.21971	0.95967
		2	0.00000	0.24494	0.19329	0.29631	0.27258	0.11779	0.95967
		3	0.00000	0.02205	0.25211	0.29643	0.27319	0.19553	0.95967
	**VUMC**	1	0.00000	0.33593	0.33644	0.38220	0.34170	0.19587	0.94710
		2	0.00000	0.28945	0.35799	0.38444	0.34812	0.33369	0.94710
		3	0.00000	0.34026	0.31697	0.38250	0.36593	0.00569	0.94710

Supplementary Table 21. **Results for meaningful identity disclosure risk**. *L* is the lower bound of the percentage of the correctly inferred attributes in a successful attack (i.e., at least L% attributes are correctly inferred).

L	Data	Run	Baseline	medGAN	medBGAN	EMR-WGAN	WGAN	DPGAN	Real
**0.1**	**UW (combined)**	1	0.00096	0.00317	0.00265	0.00353	0.00348	0.00026	0.00395
		2	0.00096	0.00097	0.00307	0.00374	0.00376	0.00027	0.00395
		3	0.00096	0.00223	0.00252	0.00357	0.00282	0.00050	0.00395
	**UW (separated)**	1	0.00096	0.00312	0.00291	0.00371	0.00287	0.00092	0.00395
		2	0.00096	0.00310	0.00205	0.00364	0.00223	0.00071	0.00395
		3	0.00096	0.00145	0.00269	0.00373	0.00208	0.00088	0.00395
	**VUMC**	1	0.00131	0.01402	0.01546	0.02359	0.01936	0.01209	0.04307
		2	0.00132	0.00960	0.01357	0.02290	0.01727	0.01631	0.04307
		3	0.00133	0.01032	0.01684	0.02528	0.02063	0.01126	0.04307
**1**	**UW (combined)**	1	0.00083	0.00178	0.00126	0.00239	0.00256	0.00015	0.00362
		2	0.00083	0.00062	0.00172	0.00251	0.00251	0.00007	0.00362
		3	0.00083	0.00065	0.00113	0.00237	0.00211	0.00027	0.00362
	**UW (separated)**	1	0.00083	0.00176	0.00139	0.00248	0.00137	0.00052	0.00362
		2	0.00083	0.00151	0.00091	0.00242	0.00100	0.00034	0.00362
		3	0.00084	0.00075	0.00132	0.00256	0.00101	0.00039	0.00362
	**VUMC**	1	0.00110	0.00282	0.00734	0.01360	0.00940	0.00618	0.03817
		2	0.00111	0.00310	0.00596	0.01357	0.01094	0.00948	0.03817
		3	0.00109	0.00582	0.00953	0.01519	0.01285	0.00554	0.03817

Supplementary Table 22. **Results for NNAA risk**.

Data	Run	Baseline	medGAN	medBGAN	EMR-WGAN	WGAN	DPGAN	Real
**UW (combined)**	1	0.00004	0.00056	0.00106	0.00709	-0.00003	0.00000	0.31693
	2	-0.00008	0.00090	0.00893	0.00637	0.00141	0.00000	0.31693
	3	0.00001	0.00780	-0.00046	0.00665	0.00246	0.00000	0.31693
**UW (separated)**	1	0.00002	0.00077	0.00909	0.00611	0.00200	-0.00233	0.31693
	2	0.00003	0.00085	0.00545	0.00545	0.00329	-0.00014	0.31693
	3	0.00006	0.00378	0.00692	0.00661	0.00281	-0.00067	0.31693
**VUMC**	1	0.00000	-0.00016	-0.00030	0.01247	0.00894	-0.00160	0.49640
	2	-0.00003	-0.00027	-0.00439	0.03011	0.00453	0.00461	0.49640
	3	-0.00005	0.00287	0.00593	0.01683	0.01190	0.00201	0.49640

Supplementary C: An example of the ranking mechanism

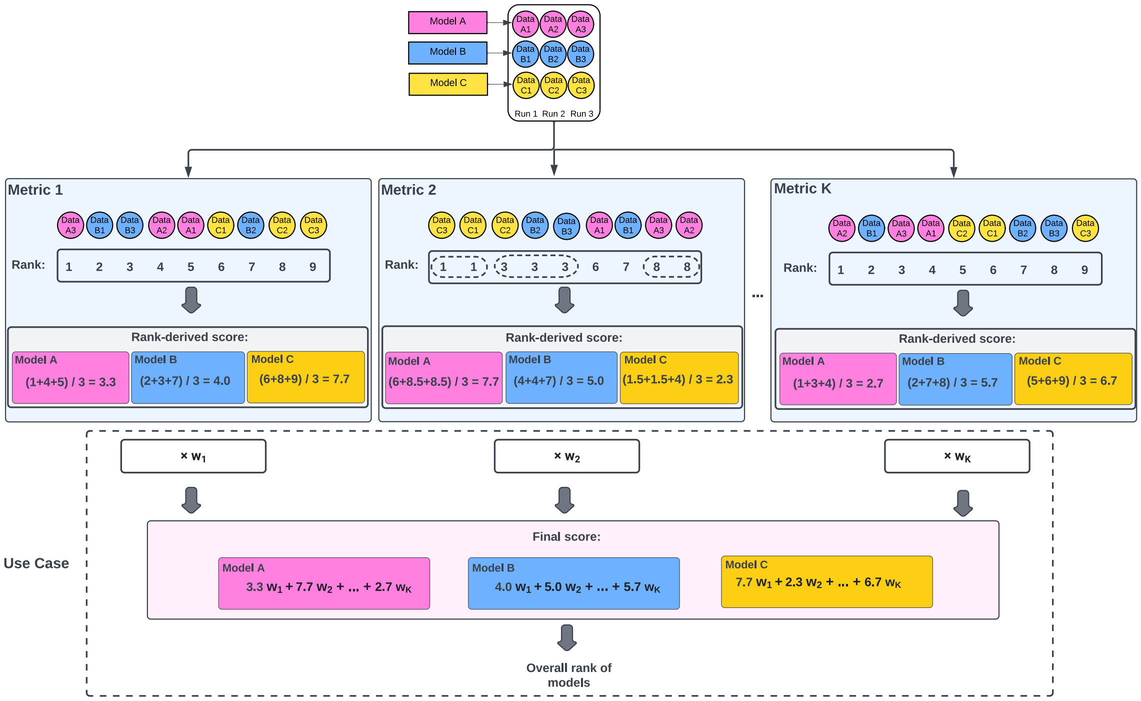

Supplementary Figure 4. **An illustration of the ranking mechanism of the benchmarking framework.** The ovals with dashed edges indicate the ties in ranks. To calculate the rank-derived score for each model regarding Metric 2, datasets C3 and C1 tied for the rank of 1 and are assigned with the same adjusted rank of 1.5 in the calculation. In the same manner, datasets C2, B2, and B3 tied for the rank of 3 and are assigned with the same adjusted rank of 4 in the calculation.

Supplementary D: Metric weight profiles for use cases.

Supplementary Table 23. **Weight profiles for individual metrics for use cases**. Note that in these use cases, we relied on TSTR results to characterize model utility in prediction performance.

**Dimensions**	**Use Cases**		
	**Education**	**Medical AI Development**	**System development**
Dimension-wise distribution	0.25	0.04	0.15
Column-wise correlation	0.15	0.04	0.04
Latent cluster analysis	0.05	0.04	0.04
Clinical knowledge violation	0.15	0.04	0.04
Medical concept abundance	0.10	0.04	0.15
Prediction performance	0.05	0.35	0.04
Feature selection	0.05	0.15	0.04
Attribute inference risk	0.05	0.075	0.125
Membership inference risk	0.05	0.075	0.125
Meaningful identity disclosure risk	0.05	0.075	0.125
NNAA risk	0.05	0.075	0.125
