# Supplementary material for: A Multifaceted benchmarking of synthetic electronic health record generation models
Source: Nat Commun. 2022 Dec 9;13:7609. doi: 10.1038/s41467-022-35295-1 (PMC9734113; doi:10.1038/s41467-022-35295-1)
Supplement: Supplementary file 2 — Reporting Summary [file 41467_2022_35295_MOESM2_ESM.pdf]

nature portfolio

Corresponding author(s): Bradley Malin, Sean Mooney

Last updated by author(s): Nov 23, 2022

Reporting Summary

Nature Portfolio wishes to improve the reproducibility of the work that we publish. This form provides structure for consistency and transparency in reporting. For further information on Nature Portfolio policies, see our Editorial Policies and the Editorial Policy Checklist.

Statistics

For all statistical analyses, confirm that the following items are present in the figure legend, table legend, main text, or Methods section.

n/a	Confirmed	
☐	☒	The exact sample size (n) for each experimental group/condition, given as a discrete number and unit of measurement
☒	☐	A statement on whether measurements were taken from distinct samples or whether the same sample was measured repeatedly
☒	☐	The statistical test(s) used AND whether they are one- or two-sided<br>*Only common tests should be described solely by name; describe more complex techniques in the Methods section.*
☒	☐	A description of all covariates tested
☒	☐	A description of any assumptions or corrections, such as tests of normality and adjustment for multiple comparisons
☐	☒	A full description of the statistical parameters including central tendency (e.g. means) or other basic estimates (e.g. regression coefficient) AND variation (e.g. standard deviation) or associated estimates of uncertainty (e.g. confidence intervals)
☒	☐	For null hypothesis testing, the test statistic (e.g. F, t, r) with confidence intervals, effect sizes, degrees of freedom and P value noted<br>*Give P values as exact values whenever suitable.*
☒	☐	For Bayesian analysis, information on the choice of priors and Markov chain Monte Carlo settings
☒	☐	For hierarchical and complex designs, identification of the appropriate level for tests and full reporting of outcomes
☐	☒	Estimates of effect sizes (e.g. Cohen's d, Pearson's r), indicating how they were calculated

Our web collection on statistics for biologists contains articles on many of the points above.

Software and code

Policy information about availability of computer code

Data collection	The electronic health record data used in this study were collected by querying 1) the UW Medicine enterprise data warehouse , and 2) the Research Derivative database at Vanderbilt University Medical Center.
Data analysis	The analysis was implemented in Python v3.7 programming environment. Other algorithms used in this study:<br>k-nearest neighbor;<br>k-means.<br><br>The source code for this study:<br>https://github.com/yy6linda/synthetic-ehr-benchmarking

For manuscripts utilizing custom algorithms or software that are central to the research but not yet described in published literature, software must be made available to editors and reviewers. We strongly encourage code deposition in a community repository (e.g. GitHub). See the Nature Portfolio guidelines for submitting code & software for further information.

Data

Policy information about availability of data

All manuscripts must include a data availability statement. This statement should provide the following information, where applicable:

- Accession codes, unique identifiers, or web links for publicly available datasets
- A description of any restrictions on data availability
- For clinical datasets or third party data, please ensure that the statement adheres to our policy

The electronic health record data that support the findings of this study are available upon request from the corresponding authors and approval from the institutions' respective IRBs. Requests for access will be processed within around 2 months subject to signing of a data use agreement.

Human research participants

Policy information about studies involving human research participants and Sex and Gender in Research.

Reporting on sex and gender	For the UW Medicine data, there are 85,490 (45.3%) male and 103,253 (54.7%) female patients included. For the Vanderbilt University Medical Center data, there are 8,990 (43.9%) male and 11,509 (56.1%) female patients included.
Population characteristics	For the UW Medicine data, there are 188,743 patients included (between January 2007 and February 2019) with 7,095 (3.8%) patients deceased within six months after their final hospital visit. For the Vanderbilt University Medical Center data, there are 20,499 COVID-19 positive patients included (between March 2020 and February 2021) with 801 (3.9%) admitted to hospital within 21 days after testing positive. See Table 1 of the main manuscript for details.
Recruitment	The participants with their mortality status indicated were identified at UW medicine. The participants with COVID-19 positive result(s) were identified at Vanderbilt University Medical Center. We extracted their electronic health record data from the data warehouses of the two medical centers.
Ethics oversight	The Institutional Review Boards at Vanderbilt University Medical Center and the University of Washington approved this study under IRB#211997 and 00011204, respectively.

Note that full information on the approval of the study protocol must also be provided in the manuscript.

Field-specific reporting

Please select the one below that is the best fit for your research. If you are not sure, read the appropriate sections before making your selection.

[x] Life sciences [ ] Behavioural & social sciences [ ] Ecological, evolutionary & environmental sciences

For a reference copy of the document with all sections, see nature.com/documents/nr-reporting-summary-flat.pdf

Life sciences study design

All studies must disclose on these points even when the disclosure is negative.

Sample size	Sample sizes were based on the availability of cases in the electronic health record databases from UW medicine and Vanderbilt University Medical Center.
Data exclusions	We did not exclude patients from the defined cohorts, but only retained features with more than 20 occurrences in each cohort.
Replication	We provided all of the intermediate results in each step as well as the analysis source code. The results of this study can be replicated through running the provided source code. The original electronic health record data require additional data request to be shared, which has been stated in the Data section.
Randomization	When building the machine learning classification models, cases were randomized into training, validation, and testing groups.
Blinding	The investigators were blinded to the group allocation during data collection and analysis.

Reporting for specific materials, systems and methods

We require information from authors about some types of materials, experimental systems and methods used in many studies. Here, indicate whether each material, system or method listed is relevant to your study. If you are not sure if a list item applies to your research, read the appropriate section before selecting a response.

Materials & experimental systems

n/a	Involved in the study	
☒	☐	Antibodies
☒	☐	Eukaryotic cell lines
☒	☐	Palaeontology and archaeology
☒	☐	Animals and other organisms
☒	☐	Clinical data
☒	☐	Dual use research of concern

Methods

n/a	Involved in the study	
☒	☐	ChIP-seq
☒	☐	Flow cytometry
☒	☐	MRI-based neuroimaging
